# *Rickettsia conorii* subsp. israelensis infection: a case report from southeast Iran

**DOI:** 10.1186/s12879-022-07291-9

**Published:** 2022-04-01

**Authors:** Mina Latifian, Mohammad Khalili, Mehrdad Farrokhnia, Ehsan Mostafavi, Saber Esmaeili

**Affiliations:** 1 Department of Epidemiology and Biostatics, Research Centre for Emerging and Reemerging Infectious Diseases, Pasteur Institute of Iran, Tehran, Iran; 2 National Reference Laboratory for Plague, Tularemia and Q Fever, Research Centre for Emerging and Reemerging Infectious Diseases, Pasteur Institute of Iran, Akanlu, KabudarAhang, Hamadan Iran; 3 Department of Pathobiology, School of Veterinary Medicine, Shahid Bahonar University of Kerman, Kerman, Iran; 4 Infectious and Tropical Research Center, Kerman University of Medical Sciences, Kerman, Iran

**Keywords:** Mediterranean spotted fever, *Rickettsia conorii*, Iran

## Abstract

**Background:**

Mediterranean spotted fever (MSF) is a zoonotic and vector-borne disease caused by *Rickettsia conorii*. We report a case (36 year-old-woman) of MSF caused by *Rickettsia conorii* from Iran.

**Case presentation:**

In September 2019, the patient was admitted to the hospital in Kerman province with flu-like symptoms and maculopapular lesions. According to the laboratory results, thrombocytopenia, elevated liver enzymes, and cardiac enzymes were observed. Skin biopsy was examined for Crimean-Congo Hemorrhagic Fever (CCHF) and MSF using the Real-Time-PCR and ELISA method. Finally, the sample was positive for *Rickettsia conorii* subsp*. israelensis* and treated with doxycycline and completely recovered.

**Conclusions:**

This study showed that MSF could be present in Iran. Therefore, identifying endemic areas in Iran for this disease should be on the agenda.

## Background

Mediterranean spotted fever (MSF) is an acute febrile and zoonotic disease caused by a Gram-negative and obligate intracellular bacterium called *Rickettsia conorii* [1]. This bacterium is a vector-borne pathogen transmitted to humans through a tick bite. *Rhipicephalus sanguineus* (brown dog tick) is the main vector for *R. conorii* [2]. MSF is geographically widely distributed and has been reported from European, African, and Asian countries. Most cases of MSF occur in the warm months of the year when the ticks are most active [3, 4].

Clinical signs of MSF are often characterized by fever, headache, maculopapular rashes, muscle pain, diarrhea, and vomiting, while all of them are nonspecific. An eschar with a black necrotic center at the site of the tick bite (called tache noire) can be found in most cases (about 75% of cases) [2]. In severe forms of the disease are seen, neurological, cardiac, and renal involvement [1]. This disease is often mild and self-limited, however, fatal cases have been reported that are similar to viral hemorrhagic fevers such as Crimean-Congo Hemorrhagic Fever (CCHF) and should be included in the differential diagnosis of a febrile syndrome with thrombocytopenia, even if tick bite and eschar are not reported [5].

MSF human cases in Iran have been reported in 2017 from Kerman province (southeast of Iran) [6]. However, it is a neglected tropical disease in Iran, and most of the clinical cases are undiagnosed. We report a first recovered patient infected with *R. conorii* subsp. *israelensis* which was diagnosed based on clinical and laboratory evidence and completely treated.

## Case presentation

The patient was a 36 year-old-woman who had no history of any underlying disease and lived in Jiroft county in Kerman province. She was a housewife and had reported no history of contact with livestock or tick bites. The initial clinical signs of the disease started on August 23th, 2019. On September 5th, 2019 the patient was admitted to Afzalpour Hospital in Kerman city with complaints of fever, chills, weakness, lethargy, muscle pain, joint pain, and bone pain. Also, she described painful dark urine, headache, and mild sore throat. At the initial examination, pulse rate, temperature, and blood pressure were 120, 36.5 °C, and 100/50 mmHg, respectively. No abnormalities were observed in clinical examination of the lung, heart, and abdomen. During the examination, the patient was conscious and mild yellowing of the eyes and diffuse skin rashes (maculopapular) were observed. The patient was hospitalized for 14 days and the progress of significant changes in her test results was as follows.

During hospitalization, a significant increase in the levels of liver enzymes (aspartate aminotransferase and alanine aminotransferase), lactate dehydrogenase and, renal markers (urea and creatinine) were observed (Table 1). The patient also had anemia and thrombocytopenia. Urine analysis showed proteinuria, hematuria, and urinary tract infections. The liver and bile ducts were normal in ultrasonography. The differential diagnosis was MSF, CCHF, and brucellosis. According to Wright and 2ME tests, the possibility of brucellosis was negative. Treatment of the patient was started with doxycycline and ceftriaxone.

**Table 1:** Laboratory findings of MSF case during hospitalization in Kerman city in 2019

Days (September)/blood analysis	5	6	7	8	9	10	11	12	14	15
White blood cell (× ${10}^{9}$/L)	6700	13,500*	15,800*	12,900*	18,300*	25,400*	25,000*	20,200*	11,700	8400
Hemoglobin (g/dl)	10.9^¥^	9.7^¥^	9.8^¥^	9.3^¥^	8.7^¥^	9.0^¥^	9.2^¥^	8.9^¥^	9.6^¥^	9.7^¥^
Platelet (× ${10}^{9}$/L)	100,000^¥^	85,000^¥^	54,000^¥^	64,000^¥^	143,000	267,000	364,000	461,000	724,000	732,000
Aspartate aminotransferase (U/L)	147*	109*	121*	99*	17	55	42	32	22	–
Alanine aminotransferase (U/L)	129*	90*	81*	62*	5	40	40	31	24	–
Alkaline phosphatase (U/L)	375*	–	332*	275	52	235	307	303	301	–
Lactate dehydrogenase (U/L)	1130*	–	–	–	–	–	–	–	–	–
Urea (mg/dl)	60*	45	30	27	50*	20	15	16	18	25
Creatinine (mg/dl)	1.7*	0.9	0.9	0.7	1.1	0.7	0.7	0.7	0.6	0.8
Ca (mg/dl)	–	3.2^¥^	4.0	3.6^¥^	–	–	4.5	–	5.2	3.6^¥^

*Increased, ^¥^Decreased

On the second day of hospitalization, the white blood cell increased from 6700 (× ${10}^{9}$/L) to 13,500 (× ${10}^{9}$/L). On the fourth day of hospitalization, partial thromboplastin time coagulation test time (PTT) was increased to more than 120 s. The white blood cell increased significantly and there was no infectious explanation for microcytic hypochromic anemia and leukocytosis. The patient’s condition did not improve with treatment. Also, temperature and blood pressures were 37.5 °C and 100/60 mmHg, respectively. To monitor the patient's condition and necessary examinations to respond to treatment, liver and kidney tests were requested daily. Blood urea and creatinine levels and electrolyte tests were abnormal and necessary treatments were performed with KCl. According to the laboratory tests, the patient had a hypocalcemia level, and treatment was started with calcium carbonate. According to the tests, the patient gradually responded to the treatment with doxycycline (100 mg twice daily) and the liver tests returned to normal and the patient’s general condition improved. Also, in hematology examinations, the leukocyte counts gradually decreased from 25,000 (× ${10}^{9}$/L) to 8400 (× ${10}^{9}$/L) and hematology service reported this was a reactionary increase.

**Fig. 1:**
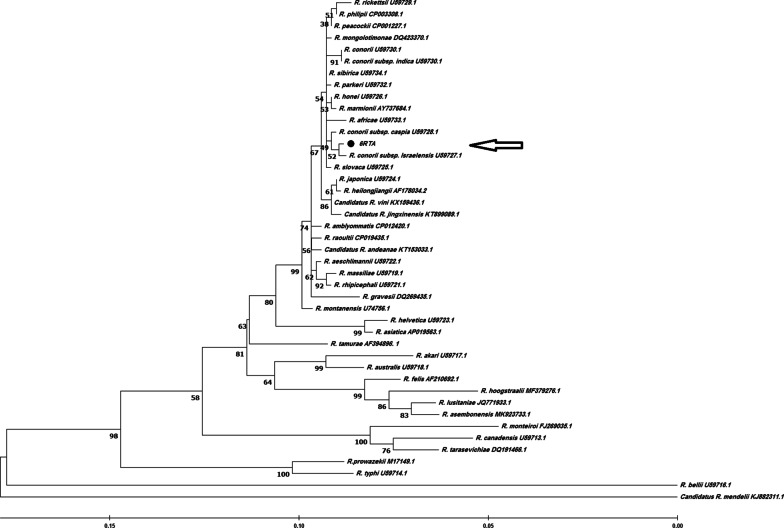
Phylogenetic analysis based on Rickettsia *gltA* gene sequencing and Maximum Likelihood method algorithm (Tamura-Nei model). The test was performed with bootstrap (500 repetitions) by MEGA X 10.1 software. Our clinical case (6RTA (GenBank Accession number ON037235)) is shown with an arrow

**Fig. 2:**
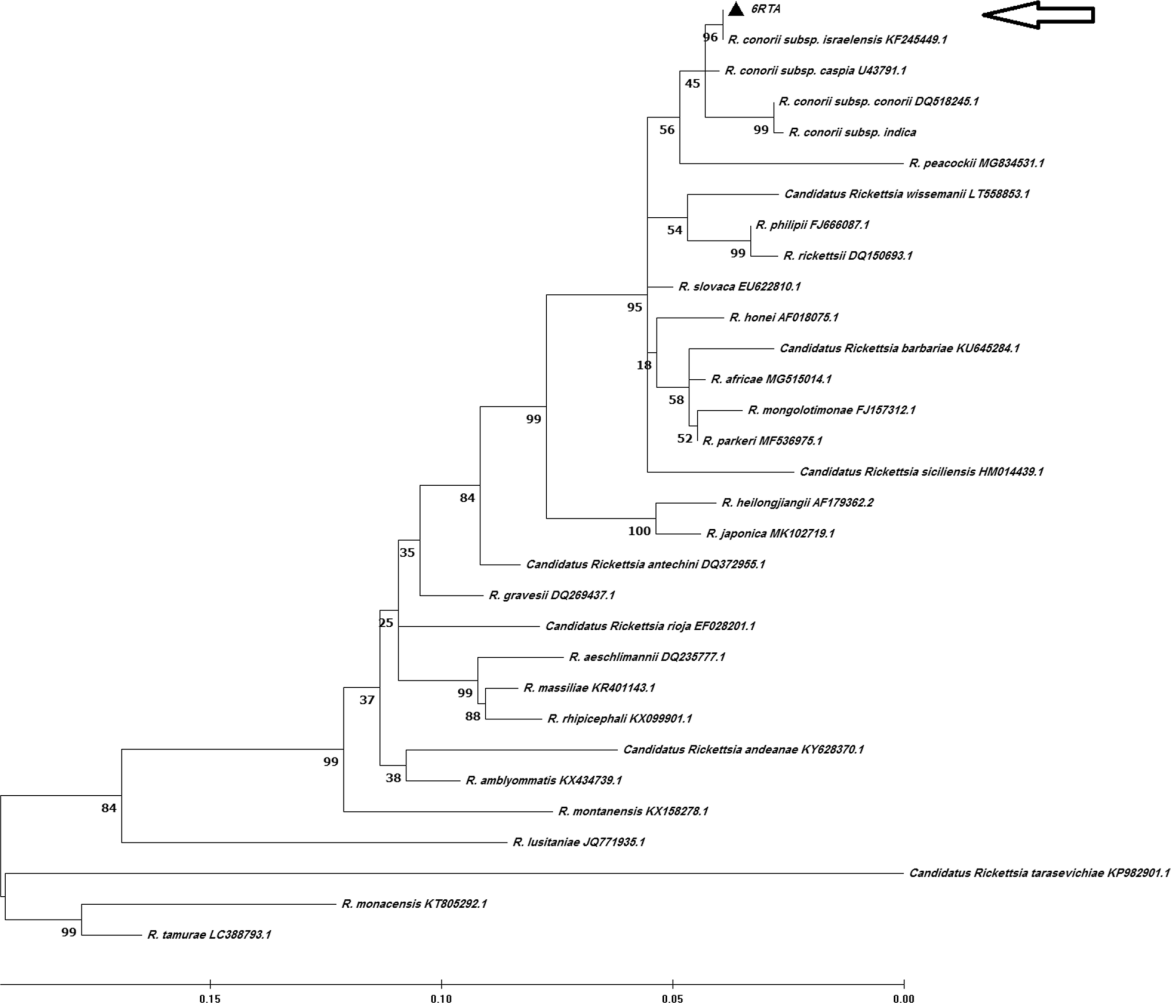
Phylogenetic analysis based on Rickettsia *ompA* gene sequencing and Maximum Likelihood method algorithm (Tamura-Nei model). The test was performed with bootstrap (500 repetitions) by MEGA X 10.1 software. Our clinical case (6RTA (GenBank Accession number ON049421)) is shown with an arrow

On the first day of admission, the skin rashes were observed in clinical examination of patient, she was diagnosed with suspected CCHF and MSF. CCHF infection was negative. Blood and tissue samples (including biopsy of skin rash) were taken from the patient and sent to the Research Centre for Emerging and Reemerging Infectious Diseases of Pasteur Institute of Iran on September 5th. The results from a *R. conorii* ELISA for detection of specific IgM antibody against *R. conorii* (Vircell co, Spain) was borderline and titer of IgM antibody against *R. conorii* was 1:48 by IFA (Vircell co, Spain). The result of real-time PCR tests (16S rRNA gene) on both biopsy of skin rash and blood samples were positive for *Rickettsia* spp (Table 2). By complementary phylogenetic studies including amplification and sequencing of specific genes of *Rickettsia* spp. including *gltA* (Fig. 1) and *ompA* (Fig. 2) and, the infection was finally confirmed as *R. conorii* subsp*. israelensis*.

**Table 2:** Primer sequences and product size used for detection and identification of *Rickettsia* spp

Gene target	Primer/probe name	Sequence (5′ to 3′)	Amplicon size (bp)	References
16S rRNA	Rsp-Forward	CGCAACCCTYATTCTTATTTGC	149	[7]
	Rsp-Reverse	CCTCTGTAAACACCATTGTAGCA		
	Rsp-probe	6- FAM-TAAGAAAACTGCCGGTGATAAGCCGGAG–TAMRA		
gltA	gltA-Forward	GCTCTTCTCATCCTATGGCTATTAT	834	[8]
	gltA-Reverse	CAGGGTCTTCRTGCATTTCTT		
ompA	ompA-Forward	ATGGCRAATATTTCTCCAAAA	632	[9]
	ompA-Reverse	GTTCCGTTAATGGCAGCATCT

On September 9th, 2019, based on the tests and clinical examinations, her condition improved and on September 17th, 2019 the symptenoms completely disappeared and the patient was discharged from the hospital on September 18th, 2019. No abnormalities were observed during follow-up after 14 days of discharge from the hospital and she was completely recovered.

## Discussion and conclusions

Here we present a recovered case of MSF, which was infected with *R. conorii* subsp. *israelensis*. The patient thought the skin rashes were appeared due to a drug injection to decrease a symptom in the previous medical center, referred to the hospital late, but after diagnosis of MSF treatment started with doxycycline and completely recovered. MSF was known as an emerging and reemerging human infection in many parts of the world [10]. Due to nonspecific clinical symptoms of MSF, diagnosis is challenging.

Several strains of *R. conorii* were identified and proposed a new species, but genotyping criteria showed that these strains belong to the species *R. conorii* because they have similar genetics but the different epidemiological and clinical expression. Finally, four subspecies of *R. conorii* including *caspia*, *israelensis*, *conorii,* and *indicia* were proposed [1]. *R. conorii* subsp. *israelensis* lead to a severe form of MSF. Due to the highest virulence of this subspecies, it can lead to unpredictable and rapidly fatal infections in elderly or immunocompromised adults [11, 12]. The geographical distribution of this disease in the Mediterranean area has already expanded. Clinical manifestations of MSF in relation to infection with *R. conorii* subsp. *israelensis* have also shown that observing tache noire was reported in 27% of cases, and also a history of tick bites is not necessarily. For this reason, sometimes the absence of tache noire delays the diagnosis and administration of antibiotics [13, 14].

The case we described did not have typical eschar and known exposure to tick. She had the initial symptoms such as flu-like symptoms, and on initial examinations, maculopapular lesions were observed. According to the laboratory results, thrombocytopenia, elevated liver enzymes, and cardiac marker were observed. The clinical manifestations of the patient in this presentation were an example of MSF and compatible with other MSF cases which reported from Zarand and Kahnuj counties (Kerman province) in 2017 [6]. Due to the spread and importance of MSF, many studies have been conducted in Iran’s neighboring countries. In one of these studies in Turkey, 11 patients with MSF were identified, and in 9 cases, molecular tests were performed on skin biopsies [15]. In this study, a skin biopsy sample was used for molecular testing, which is one of the best samples for diagnosing MSF [16]. MSF should be considered as a differential diagnosis in patients with fever who have symptoms of skin rash with thrombocytopenia, elevated liver enzymes, and impaired renal function.

MSF is referred to as a seasonal disease because it is associated with the biological activity of ticks. Laboratory evidence suggests that changes in temperature are associated with changes in the tendency of ticks to humans [17]. These studies show that the tendency of ticks to human bites has increased at warmer temperatures because ticks are more active during this period, so it is predicted that more pathogens will emerge through ticks due to global warming. Raising awareness of MSF, especially in endemic areas, is very important in order to control the disease, and identifying endemic areas in Iran for this disease should be on the agenda.

## Data Availability

All data generated or analysed during this study are included in this published article.

## Abbreviations

MSF: Mediterranean spotted fever
CCHF: Crimean-Congo hemorrhagic fever
PTT: Partial thromboplastin time coagulation test time
